# The role of plasma gelsolin in cardiopulmonary bypass induced acute lung injury in infants and young children: a pilot study

**DOI:** 10.1186/1471-2253-14-67

**Published:** 2014-08-07

**Authors:** ShanShan Shi, Chi Chen, DongYan Zhao, XiWang Liu, BaoLi Cheng, ShuiJing Wu, Ru Lin, LinHua Tan, XiangMing Fang, Qiang Shu

**Affiliations:** 1 Surgical Intensive Care Unit, Children’s Hospital, Medical College, Zhejiang University, No. 57, Zhugan Lane, Hangzhou 310003, P R China; 2 Department of Thoracic & Cardiovascular Surgery, Children’s Hospital, Medical College, Zhejiang University, and Zhejiang Key Laboratory for Diagnosis and Therapy of Neonatal Diseases, No. 57, Zhugan Lane, Hangzhou 310003, P R China; 3 Department of Anesthesiology, the First Affiliated Hospital, Medical College, Zhejiang University, No. 79, Qingchun Road, Hangzhou 310003, P R China

**Keywords:** Plasma gelsolin, Acute lung injury, Cardiac surgery, Cardiopulmonary bypass, Infant, Young children, Congenital heart disease

## Abstract

**Background:**

Acute lung injury (ALI) induced by cardiopulmonary bypass (CPB, CPB-ALI) is a common and serious complication after cardiac surgery. And infants and young children are more prone to CPB-ALI. The purpose of this study was to investigate the perioperative changes of plasma gelsolin (pGSN) in patients below 3years of age with cardiac surgeries and CPB, and determine whether pGSN are associated with the occurrence and severity of CPB-ALI.

**Methods:**

Seventy-seven consecutive patients ≤3 years of age with congenital heart diseases (CHD) performed on open heart surgery with CPB were finally enrolled, and assigned to ALI and non-ALI groups according to the American-European Consensus Criteria. Plasma concentrations of gelsolin and total protein were measured at following 8 time points: before CPB (a), after CPB (b), 2 hours after CPB (c), 6 hours after CPB (d), 12 hours after CPB (e), 24 hours after CPB (f), 48 hours after CPB (g) and 72 hours after CPB (h).

**Results:**

Twenty-seven (35.1%) patients developed CPB-ALI in the study, including eleven (14.3%) patients with ARDS. The earliest significant drop of pGSN and normalized pGSN (pGSN_N_) of ALI group both occurred at 6 hours after CPB (*p* = 0.04 and *p* < 0.01), which was much earlier than those of non-ALI group (48 hours, *p* = 0.03 and 24 hours, *p* < 0.01); PGSN of ALI group before CPB and 6 hours after CPB were both significantly lower than those of non-ALI group (*p* < 0.01); PGSN_N_ of ALI group before CPB and 6 hours after CPB were both significantly lower than those of non-ALI group (*p* < 0.01, *p* = 0.04); PGSN before CPB was the only independent risk factor predicting the occurrence of CPB-ALI (OR, 1.023; 95% CI, 1.007-1.039; *p* < 0.01) with an AUC of 0.753 (95% CI, 0.626-0.880); The optimal cutoff value of pGSN before CPB was 264.2 mg/L, with a sensitivity of 58.3% and a specificity 94.7%. And lower pGSN before CPB was significantly associated with the severity of CS-AKI (*r* = −0.45, *p* < 0.01).

**Conclusions:**

Patients developing CPB-ALI had lower plasma gelsolin reservoir and a much more amount and rapid consumption of plasma gelsolin early after operation. PGSN before CPB was an early and sensitive predictor of CPB-ALI in infants and young children undergoing cardiac surgery, and was negatively correlated with the severity of CPB-ALI.

## Background

Acute lung injury induced by cardiopulmonary bypass, a common and serious complication in patients undergoing cardiac surgery necessitating CPB, may impair both lung mechanics and gas exchange, and becomes an important factor of morbidity and mortality after surgery [1,2]. 2% of those patients may develop into acute respiratory distress syndrome (ARDS) resulting in a mortality of 15% to 50% [3]. Infants and children are more prone to CPB-ALI which is associated with postoperative pulmonary complications, prolonged mechanical ventilation, as well as longer ICU time and hospital resource utilization [4,5]. Therefore, there is increasing interest in developing some effective biomarkers for prediction of CPB-induced ALI in infants and children, which would be helpful for early diagnosis and decision-making.

Gelsolin, a protein of 82-84KDa, is the founding member of gelsolin protein superfamily, as a cytoplasmic as well as a plasma isoform, can bind, sever and cap actin filaments. Plasma gelsolin, primarily secreted by myocytes, is believed to function as an important actin scavenger in the circulation [6-8]. Besides, emerging data have demonstrated that pGSN, as an inflammatory mediator-binding protein, also functions as a lipid carrier with strong binding affinity to several plasma lipids such as lipopolysaccharide exdotxin, lipoteichoic acid, lysophosphatidic acid, β-amyloid peptide and platelet-activating factor, etc., and plays a role in localizing inflammation and prevent systemic escape of pro-inflammatory lipids [9-12]. Consistent with these proposed functions, declining pGSN level has be proved to be associated with a variety of acute clinical conditions, such as sepsis, myocardial infarction, major trauma, burn, allogeneic stem cell transplantation and acute liver injury, etc. And more importantly, pGSN levels are inversely related to the clinical outcome under these conditions [13-18].

For systemic inflammatory response syndrome (SIRS) is the origin of CPB-ALI, we speculated that pGSN might play a role in the development of CPB-ALI. The purpose of this study was to investigate the perioperative changes of pGSN level in patients below 3 years of age undergoing cardiac surgeries with CPB, and determine whether pGSN levels are associated with the occurrence and severity of CPB-ALI.

## Methods

### Protocol

The study was conducted in a 20-bed Surgical ICU (SICU) from November 2012 to March 2013 at the children’s hospital, Medical College, Zhejiang University, P. R. China. The present study was approved by local hospital ethics committee (Medical Ethics Committee, Children’s Hospital, Medical College, Zhejiang University), and written informed consents were obtained from their parents. Seventy-seven consecutive patients aged ≤3 years (including 3 neonates) with congenital heart diseases undergoing open heart surgery with CPB were finally enrolled into the study. Patient with pneumonia, liver/renal dysfunction, major chromosomal abnormalities, need for mechanical ventilation before surgery, premature babies, or low cardiac output syndrome after surgeries was excluded from the study.

Anesthesia and CPB procedure were managed according to the standard protocol. Myocardial protection was achieved using cold blood cardioplegic solution unless otherwise indicated. The patients were transferred to SICU immediately after surgery. Each patient was monitored continuously for hemodynamic and respiratory data. Blood gas analysis was performed routinely pre-, intra-, and post-operation. All the patients admitted to SICU were ventilated with initial inspiratory oxygen fraction (FiO_2_) 50% and initial positive end expiratory pressure (PEEP) 3 cmH_2_O, and then adjusted to maintain normocapnia and oxygen saturation (SaO_2_) higher than 95% with lowest FiO_2_ and most appropriate PEEP. And chest radiograph was performed every 24 hours.

The criteria of weaning from invasive ventilation in our unit are provided in detail in Additional file 1. Noninvasive ventilation (continuous positive airway pressure, CPAP) was used immediately after thacheal extubation in patients at high risk of extubation failure (EF) (Additional file 1), or started when acute respiratory failure appears after extubation. The initial CPAP setting was accorded to the each patient’s age and weight, and adjusted by the clinical status. Patients not responding successfully to CPAP were reintubated according to the criteria provided in Additional file 1, and the decision was made by the attending physicians.

### Definition of CPB-ALI

Patients were categorized into CPB-ALI group if they met the American-European Consensus Criteria within 24 h after surgery [19]. The diagnosis was made by the physician blind to the study.

### Data and blood sampling collection

Whole blood samples (3 ml) were obtained from patients at following time points: before CPB (a), after CPB (b), 2 hours after CPB (c), 6 hours after CPB (d), 12 hours after CPB (e), 24 hours after CPB (f), 48 hours after CPB (g) and 72 hours after CPB (h), and were collected in EDTA-containing tubes. After being centrifuged at 2500 g for 5 minutes at 4°C, plasma samples were frozen at −80°C until analysis. PGSN levels were measured using an ELISA kits (USCN, Wuhan, P. R. China) according to the manufacturer’s instruction. The plasma total protein amount (TP) was measured at each time point by an auto-biochemistry analyzer (Modular Analytics, Roche, USA) for pGSN normalization.

The clinical variables included sex, age, age distribution, weight, risk adjustment for surgery for congenital heart disease (RACHS-1), CPB time, aortic cross-clamp time, nadir HCT (%) during CPB, ultrafiltrated volume removal (UVR), positive fluid balance after CPB, need for transfusion within 24 hours after CPB, nosocomial pneumonias (NF), extubation failure (EF), as well as mechanical ventilation duration, need for CPAP, ICU stay, hospital stay and hospital death. The definitions for the clinical variables are provided in Additional file 2.

### Statistical analysis

Continuous variables were tested for normal distribution by one-sample Kolmogorov-Smirnov test, and were expressed as mean ± SD if normally distributed, and otherwise, as median (interquartile range). Student’s *t*-test or the Mann–Whitney *U*-test was used to determine the significant difference of continuous variables between two groups; χ^2^ test was used for the comparison of categorical variables. The both changes of pGSN and pGSN_N_ (pGSN/TP ratio, 10^−3^) level over times of each group were analyzed by two-way ANOVA, and an analysis of covariance procedure (ANCOVA) was used to test the differences of both pGSN and pGSN_N_ between two groups. Logistic regression analysis was used to identify the independent risk factors for CPB-ALI. The preoperative, intraoperative and early postoperative variables with a *p* value less than 0.1 were entered into the logistic model, including age, age distribution, weight, positive fluid balance after CPB, TP and pGSN before CPB. Area under the ROC curve was used to assess how well the factors diagnose CPB-ALI. A Pearson or Spearman correlation test was performed to determine the correlation between pGSN and severity of CS-AKI and other clinical outcomes. A *p* value less than 0.05 was considered to be statistically significant. Statistical analysis was performed using SPSS 16.0 (SPSS Inc, Chicago, IL, USA) software version.

## Results

### Clinical outcomes

During the study period, a total of 77 patients aged ≤ 3 years were enrolled in the study. Among the 77 patients, 27 (35.1%) developed CPB-ALI, including eleven (14.3%) patients with ARDS. As shown in Table 1, no differences were observed in gender distribution, RACHS-1 score, CPB time, aortic cross-clamp time, nadir HCT during CPB, UVR, need for transfusion within 24 hours after CPB, EF or NF incidence and hospital stay between the two groups. However, compared with patients without CPB-ALI, the patients with CPB-ALI were significantly younger in age, especially in patients <1 year of age, lighter in weight, higher incidence of positive fluid balance after CPB. In addition, the durations of mechanical ventilation and ICU stay of ALI group were both longer than those of non-ALI group; the patients with CPB-ALI were more likely to require CPAP after thacheal extubation. No patients died in hospital in this pilot study.

**Table 1:** Demographic and clinical characteristics of the patients enrolled in the study

	**Total**	**ALI (n = 27)**	**Non-ALI (n = 50)**	***p***
Sex male n (%)	55(71.4)	17(63.0)	38(76.0)	0.27^C^
Age (months)	9.1 ± 6.8	5.8 ± 4.5	10.9 ± 7.2	0.001^a^
Age distribution				0.03^C^
0-1 year old	54	23(42.6)	31(57.4)	
1-2 years old	19	4(21.1)	15(78.9)	
2-3 years old	4	0(0)	4(100)	
Weight (kg)	7.3 ± 2.2	6.0 ± 1.8	8.0 ± 2.1	0.001^a^
RACHS-1				0.37^C^
1	10	3	7	
2	63	21	42	
≥3	4	3	1	
CPB time (min)	69.4 ± 23.3	74.6 ± 22.1	66.7 ± 23.7	0.23^a^
Aortic cross clamp time (min)	50.1 ± 19.6	46.5 ± 17.7	44.2 ± 20.7	0.68^a^
Nadir HCT during CPB (%)	25.3 ± 2.7	25.2 ± 2.3	25.2 ± 3.0	0.98^a^
Positive fluid balance after CPB (%)	55(71.4)	24(88.9)	31(62.0)	0.03^C^
Need for transfusion within 24 hours after CPB (%)	6(7.8)	2(7.4)	4(8.0)	0.93^C^
Nosocomial pneumonias (%)	15(19.5)	6(22.2)	9(18.0)	0.58^C^
CPAP (%)	10(13.0)	9(33.3)	4(8.0)	0.04^C^
EF (%)	3(3.9)	3(11.1)	0(0)	0.12^C^
MV time (hour)	24(3 ~ 446)	26(4 ~ 446)	6.5(3 ~ 189)	0.001^b^
SICU stay (day)	5(2 ~ 34)	9.2 ± 6.6	4(2 ~ 34)	0.001^b^
Hospital stay (day)	23.4 ± 10.0	26.1 ± 11.0	21.9 ± 9.2	0.15^a^
Hospital death (%)	0	0	0	0.77^C^

Continuous variables were expressed as the mean ± SD or median (interquartile range) if appropriated. Categorical variable were expressed as number of patients (%).

^a^: Student’s *t*-test; ^b^: Mann–Whitney *U*-test; ^C^: χ^2^ test.

RACHS-1 = Risk Adjusted Classification for Congenital Heart Surgery; ALI = acute lung injury; CPB = cardiopulmonary bypass; MV = mechanical ventilation; CPAP = Continuous positive airway pressure; EF = Extubation failure; SICU = surgery intensive care unit; UVR = ultrafiltered volume removal.

### The perioperative changes of plasma gelsolin and total protein

**Figure 1:**
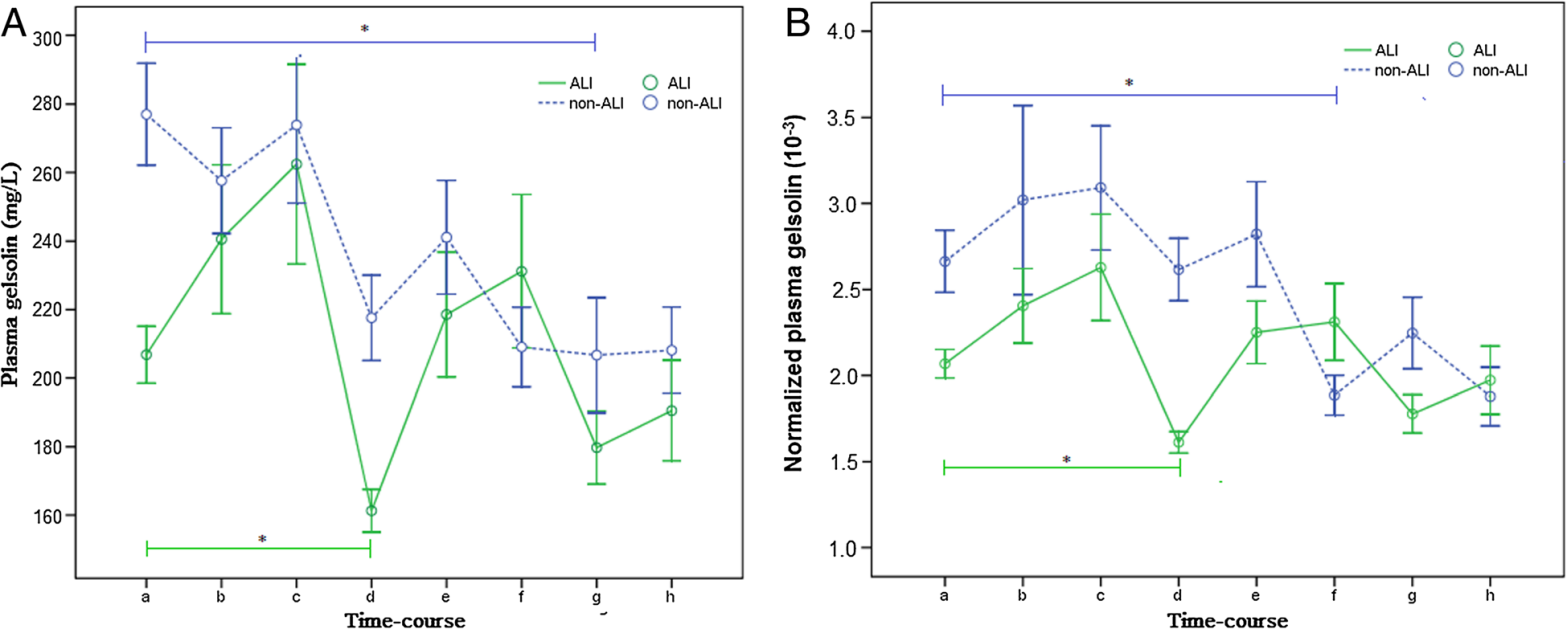
**The time course of plasma gelsolin. A**: Perioperative change of pGSN levels in ALI (green line) and non-ALI groups (blue line); **B**: Perioperative change of pGSN_N_ levels in ALI and non-ALI groups; *Two-way ANOVA, *p* < 0.05 versus before CPB (baseline level).

Plasma gelsolin levels were showed in Table 2, both as absolute value (pGSN) and normalized value (pGSN_N_). For the plasma total protein level after CPB decreased dramatically (*p* < 0.01), both pGSN and pGSN_N_ levels were used for univariate analysis. In the ALI group, both of the earliest significant drop of pGSN and pGSN_N_ occurred at 6 hours after CPB compared with those of the preoperative levels (baseline level, *p* = 0.04 and *p* < 0.01), while the first remarkable drops of pGSN and pGSN_N_ in non-ALI group occurred at 48 hours after CPB (*p* = 0.03) and 24 hours after CPB (*p* < 0.01) respectively (Figure 1).

**Table 2:** Perioperative changes of plasma gelsolin and total protein levels

	**Total**	**ALI (n = 27)**	**Non-ALI (n = 50)**	***p***
Before CPB (a)				
TP (g/L)	62.4 ± 5.6	59.3 ± 5.6	64.1 ± 4.8	0.01^a^
pGSN (mg/L)	157.8 ± 52.8	122.6 ± 24.6	176.4 ± 54.5	0.01^a^
pGSN_N_ (10^−3^)	2.51 ± 0.84	2.02 ± 0.39	2.77 ± 0.89	0.01^a^
After CPB (b)				
TP (g/L)	55.1 ± 4.6	55.3 ± 4.9	55.0 ± 4.7	0.81^a^
pGSN (mg/L)	138.7 ± 51.9	133.2 ± 54.0	141.7 ± 51.2	0.53^a^
pGSN_N_ (10^−3^)	2.52 ± 0.92	2.41 ± 0.95	2.33 ± 0.98	0.11^a^
2 hours after CPB (c)				
TP (g/L)	56.6 ± 5.2	54.9 ± 4.9	57.5 ± 5.1	0.08^a^
pGSN (mg/L)	152.0 ± 73.7	142.9 ± 66.9	156.8 ± 77.6	0.65^a^
pGSN_N_ (10^−3^)	2.33(1.26 ~ 0.76)	2.62 ± 1.24	2.747 ± 1.35	0.23^a^
6 hours after CPB (d)				
TP (g/L)	56.9 ± 5.8	55.6 ± 6.6	57.6 ± 4.9	0.22^a^
pGSN (mg/L)	124.3 ± 47.8	89.9 ± 17.6	141.0 ± 49.0	0.01^a^
pGSN_N_ (10^−3^)	1.80(1.15 ~ 4.63)	1.61 ± 0.26	2.18 ± 0.75	0.04^a^
12 hours after CPB (e)				
TP (g/L)	58.0 ± 4.6	55.7 ± 3.7	59.1 ± 4.7	0.01^a^
pGSN (mg/L)	135.9 ± 54.0	121.3 ± 38.3	142.5 ± 59.1	0.33^a^
pGSN_N_ (10^−3^)	2.34 ± 0.91	2.19 ± 0.73	2.41 ± 0.98	0.40^a^
24 hours after CPB (f)				
TP (g/L)	58.1 ± 5.4	57.7 ± 4.8	58.3 ± 5.7	0.71^a^
pGSN (mg/L)	124.6 ± 43.9	133.0 ± 58.2	121.0 ± 36.5	0.37^a^
pGSN_N_ (10^−3^)	2.16 ± 0.75	2.31 ± 0.87	2.09 ± 0.69	0.98^a^
48 hours after CPB (g)				
TP (g/L)	60.1 ± 5.8	61.3 ± 5.3	60.4 ± 6.2	0.61^a^
pGSN (mg/L)	123.1 ± 39.8	111.7 ± 27.0	129.5 ± 44.6	0.14^a^
pGSN_N_ (10^−3^)	2.05 ± 0.74	1.80 ± 0.45	2.19 ± 0.83	0.33^a^
72 hours after CPB (h)				
TP (g/L)	64.4 ± 7.2	66.2 ± 7.7	63.1 ± 6.6	0.18^a^
pGSN (mg/L)	131.2 ± 39.6	124.6 ± 37.8	136.4 ± 41.3	0.33^a^
pGSN_N_ (10^−3^)	2.02 ± 0.63	1.92 ± 0.55	2.08 ± 0.67	0.31^a^

Data were expressed as the mean ± SD or median (interquartile range) if appropriated.

^a^: ANCOVA.

TP = plasma total protein amount; pGSN = plasma gelsolin; pGSN_N_ = plasma gelsolin normalized by plasma total protein amount.

**Figure 2:**
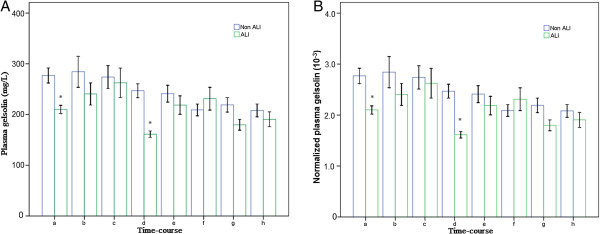
**The comparison of plasma gelsolin between two groups. A**: The comparison of pGSN levels between two groups at each time point; **B**: The comparison of pGSN_N_ levels between two groups at each time point; *ANCOVA, *p* < 0.05.

The pGSN level of ALI group before CPB and 6 hours after CPB were (122.6 ± 24.6) mg/L and (89.9 ± 17.6) mg/L, which were significantly lower than those of non-ALI group [(176.4 ± 54.5) mg/L, *p* < 0.01 and (141.0 ± 49.0) mg/L, *p* < 0.01]. Likewise, the pGSN_N_ of ALI group before CPB and 6 hours after CPB were (2.02 ± 0.39) × 10^−3^ and (1.61 ± 0.26) × 10^−3^, which were also significantly lower than those of non-ALI group [(2.77 ± 0.89) × 10^−3^, *p* < 0.01 and (2.18 ± 0.75) × 10^−3^, *p* = 0.04] (Table 2, Figure 2).

The TP level before surgery of ALI group was significantly lower than that of non-ALI group [(59.3 ± 5.6) *vs.* (64.1 ± 4.8), *p* < 0.01].

### Low plasma gelsolin before CPB as a predictor for CPB-ALI

**Figure 3:**
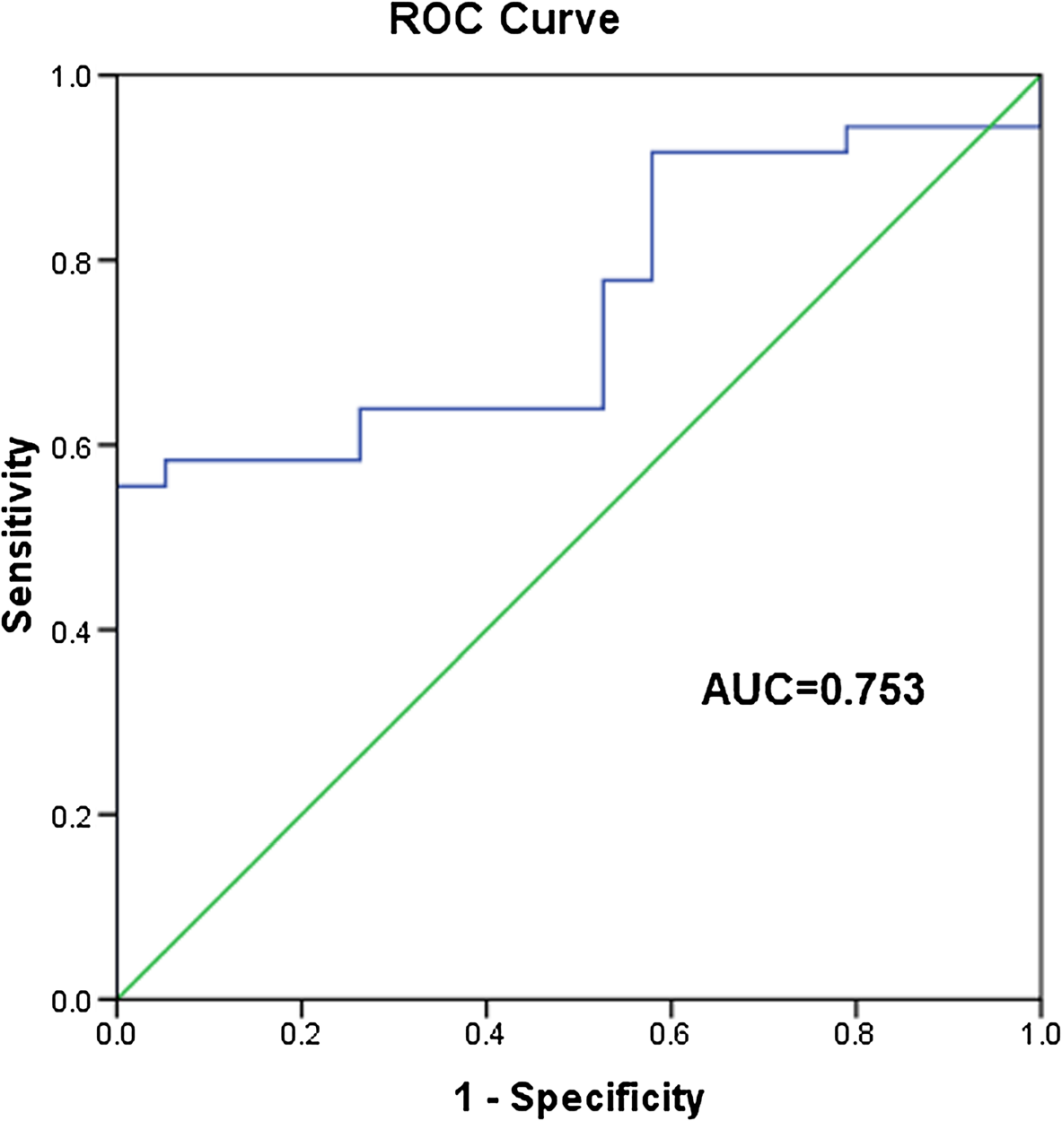
**ROC curve for pGSN before CPB.** The AUC of pGSN before CPB for CPB-ALI was 0.753 (95%CI, 0.626 to 0.880).

Logistic regression revealed that pGSN before CPB was the only independent risk factor for predicting the occurrence of CPB-ALI (OR, 1.023; 95% CI, 1.007-1.039; *p* < 0.01) among the six factors (age, age distribution, weight, positive fluid balance after CPB, TP and pGSN before CPB). In the ROC analysis (Figure 3), the AUC of pGSN before CPB was 0.753 (95% CI, 0.626-0.880). And the optimal cutoff for pGSN before CPB was 264.2 mg/L, with a sensitivity of 58.3% and a specificity 94.7%.

### Plasma gelsolin predict the severity of CPB-ALI

**Figure 4:**
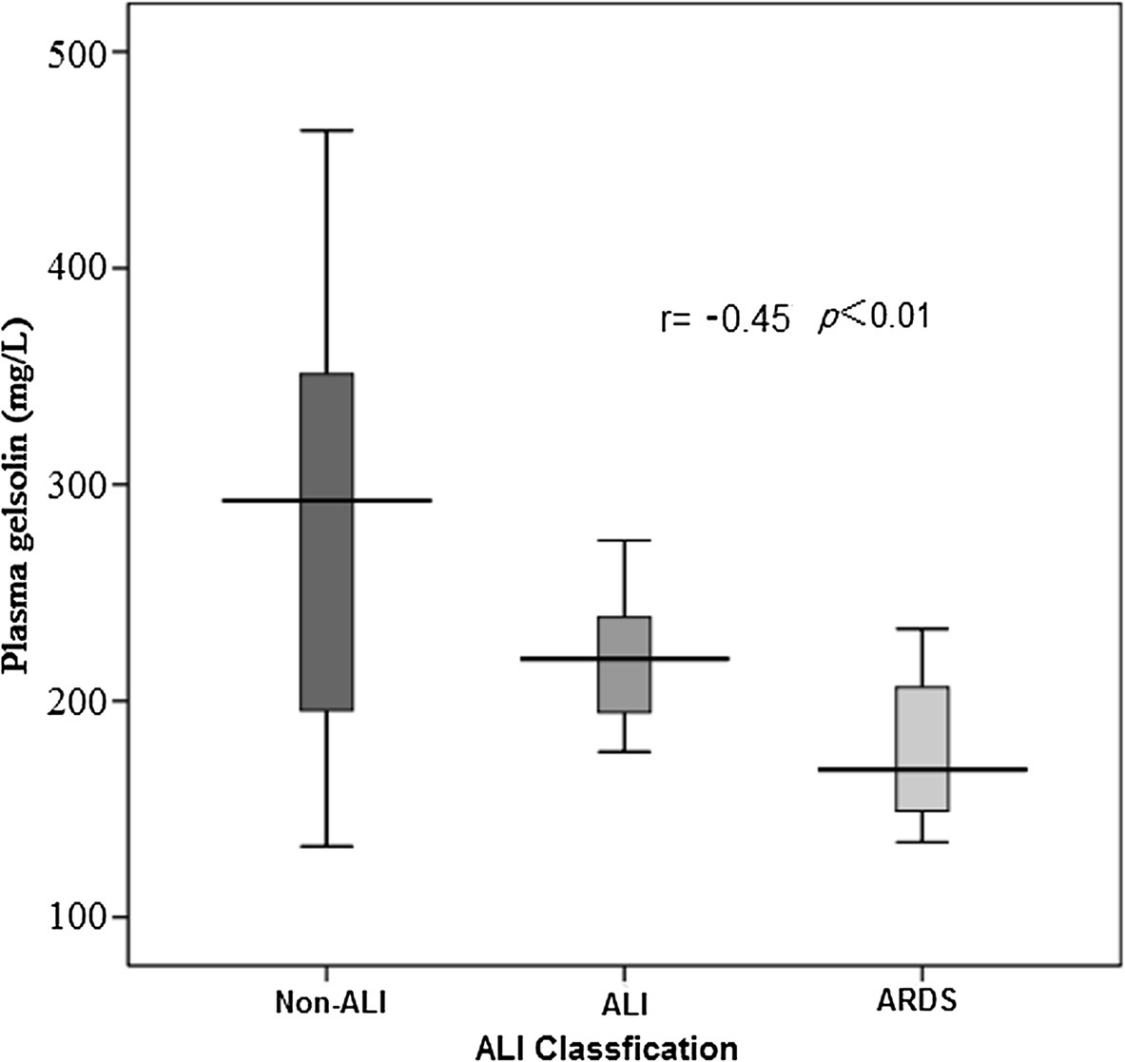
**The severity of CPB-ALI.** PGSN before CPB was significantly negative correlated with the severity of CPB-ALI in this study (*r* = −0.45, *p* < 0.01).

Lower pGSN before CPB was significantly associated with the severity of CPB-ALI in this study (Figure 4, *r* = −0.45, *p* < 0.01). Though age, age distribution, weight, positive fluid balance after CPB and TP before CPB were excluded from final multiple regression analysis, younger age, patients ≤1 year of age, lighter weight and low total protein level before CPB were proved to be significantly correlated with the severity of CPB-ALI (*r* = −0.335, *p* < 0.01; *r* = −0.383, *p* < 0.01; *r* = −0.441, *p* < 0.01 and *r* = −0.395, *p* < 0.01).

## Discussion

The lungs are proposed to be one of most “sensitive” organ to the SIRS, especially in pediatric population [20]. The incidence of CPB-ALI of this cohort was 35.1%, and 14.3% of patients developed ARDS, which was much higher than that of adult population. In univariate analysis, we found that weight was much lower in ALI group, and they were much younger than patients without ALI. The patients’ weight and age of this study were significantly correlated with the severity of CPB-ALI (*r* = −0.441, *p* < 0.01; and *r* = −0.335, *p* < 0.01). And the patients below 1 year old were especially prone to CPB-ALI compared with elder children (42.6% vs. 21.1%), and was also strongly correlated with the severity of CPB-ALI (*r* = −0.383, *p* < 0.01). Furthermore, we also demonstrated that the preoperative plasma protein level of ALI group was much lower than that of non-ALI group (*p* < 0.01), and patients with ALI were vulnerable to positive fluid balance after CPB (*p* = 0.03). So, it seems that bronchopulmonary immaturity, hypoproteinemia, additional fluid retention worsens its inability to adapt the environment of systemic inflammatory response provoked by CPB, which might consequently aggravate the increase of permeability, pulmonary vascular resistant and the changes of lung surfactant, and exacerbate lung disturbances early after surgery [2,3,21]. Though transfusion of blood products during or after surgery had been proved to be a strong risk factor of acute lung injury and might associated with increased mortality in long-term follow-up [22-24], there were no significant differences of nadir HCT during CPB and incidence of transfusion early after surgery between two groups in our study. We proposed that the possible reasons might be the restrictive transfusion policy both in CPB circuit priming (only in patients with an estimated nadir HCT ≤25% during CPB) and after surgery (only in patients with Hb ≤ 7 g/dL or coagulation dysfuction), and routine use of ultrafiltration and CPB circuit residual blood salvage re-infusion in all the patients. Consistent with other age-match population, the patients with CPB-ALI of this cohort did need longer mechanical ventilation and ICU-stay time than patients without ALI, which may related to addition lung fluid retention, delayed recovery of ability of gas exchange and lung mechanics, infectious or noninfectious pulmonary complications, susceptibility to low cardiac output syndrome and other institutional and practice guidelines [25,26]. The prognosis of patients with CPB-ALI in this study was good without hospital death and with similar duration of hospital stay in both groups.

For the systemic inflammatory response was proposed to play a pivotal role in the pathogenesis of CPB-induced acute lung injury [27,28], various of factors concerned inflammation, not only the changes in the levels, but also the time courses or the patterns of release, had been described for the affecting the incidence, severity and clinical outcome of the CPB-ALI such as IL-6, IL-8, TNF-α and endotoxins et al. Recent studies highlighting the novel early biomarks of CPB-ALI were performed, such as surfactant derived protein B (SPB) and plasma receptor for advanced glycation end products (RAGE), and have shown to be a promising diagnostic method in both adult and pediatric population [29,30]. Though, the change of pGSN in acute lung injury was still controversial for the differences of etiologies, study kinetic and damage severity. As a novel inflammatory mediator, declining pGSN had demonstrated its predictor and therapeutic value in several acute lung injury model related to SIRS, such as sepsis, burn and trauma [31-33]. In this study, we observed the time course characteristics of pGSN of patients undergoing open heart surgery with CPB, and its role in predicting the occurrence and severity of CPB-ALI.

The total baseline level of pGSN (preoperative level) in this study was (157.8 ± 52.8) mg/L, which was much lower than of that of adult population of 190-300 mg/L [34]. And the baseline level of pGSN of ALI patients was significantly lower than that of patients without CPB-ALI [(122.6 ± 24.6) mg/L *vs.* (176.4 ± 54.5) mg/L, *p* < 0.01]. This meant that patients with lower plasma gelsolin “reservoir” were more prone to acute lung injury after CPB, and lower preoperative pGSN level might be a possible early predictor of CPB-ALI [15-18,35]. In addition, for recombinant pGSN is available, gelsolin supplement could be a safe and promising therapy in preventing and alleviating CPB-ALI before surgery in the future. We also found that both pGSN and pGSN_N_ of ALI group decreased significantly at 6 hours after CPB, which were much earlier than those of non-ALI group (48 and 24 hours after CPB); PGSN and pGSN_N_ of ALI group at 6 hours after CPB were much lower than those of non-ALI group. The depletion of pGSN was due to the decreased production and increased consumption. Though the production of gelsolin could not be identified for the difference of individuals’ muscle mass, we speculated a much more amount and rapid consumption of plasma gelsolin in ALI group for two main reasons. First, pGSN sequestration at lesions or clearance with circulating actin released from damaged and dying cells or tissues is a principal reason for depletion of pGSN. Second, as an inflammatory mediator-binding protein in the circulation, pGSN binds to the quantities of inflammatory mediator and pro-inflammatory cytokines provoked by CPB, as to alleviate the local and systemic inflammatory response.

After collecting all the possible risk factors into final multiple regression analysis, we found that pGSN before CPB was the only independent risk factor predicting for CPB-ALI (OR, 1.023; 95% CI, 1.007-1.039; *p* < 0.01), with an AUC of 0.753 (95% CI, 0.626-0.880); The optimal cutoff for pGSN before CPB was 264.2 mg/L, with a sensitivity of 58.3% and a specificity 94.7%. And lower pGSN before CPB was significantly associated with the severity of CPB-ALI in this study (Figure 4, *r* = −0.45, *p* < 0.01). These findings demonstrated that pGSN before CPB could not only be a novel early biomarker for CPB-ALI, but also a reliable quantitative factor reflecting the severity of CPB-ALI in infants and young children.

### Limitation

Limitation of the present study should be acknowledged. First, the study was based on a single medical center, local practice patterns, and a small number of patients, which might impede the application of present findings to other institutions. Second, the population of the study was restricted in the patients with CHD below 3-year-old, so we could not be sure whether these findings were available for elder children or adult patients. Third, the gelsolin level in the lung tissue was not detected in the study, therefore, the correlation of gelsolin between site of injury and circulation was still unclear.

## Conclusions

Patients developing CPB-ALI had lower plasma gelsolin reservoir and a much more amount and rapid consumption of plasma gelsolin early after operation. PGSN before CPB was an early and sensitive predictor of CPB-ALI in infants and young children undergoing cardiac surgery with CPB, and was negatively correlated with the severity of CPB-ALI.

## Competing interests

The authors declare that they have no competing interests.

## Authors’ contributions

SSS participated in the design of the study and drafted the manuscript. CC and SJW collected the clinical data and blood samples of the patients, and helped to draft the manuscript. DYZ carried out the statistical analysis and helped to draft the manuscript. BLC and XWL carried out the ELISA test. RL and LHT participated in the design of the study and coordination and helped to draft the manuscript. QS and XMF participated in the design of the study and critical revision of the article for the important content. All authors had read and approved the final manuscript.

## Pre-publication history

The pre-publication history for this paper can be accessed here:

http://www.biomedcentral.com/1471-2253/14/67/prepub

## Supplementary Material

Additional file 1 Guideline for respiratory managements. Click here for file

Additional file 2 Definitions of clinical variables. Click here for file
