# Supplementary material for: The role of plasma gelsolin in cardiopulmonary bypass induced acute lung injury in infants and young children: a pilot study
Source: BMC Anesthesiol. 2014 Aug 7;14:67. doi: 10.1186/1471-2253-14-67 (PMC4132929; doi:10.1186/1471-2253-14-67)
Supplement: Additional file 1 — Guideline for respiratory managements. [file 1471-2253-14-67-S1.doc]

**Additional File 1: Guideline for respiratory managements**

**Extubation Criteria**

- Stable hemodynamic profile, normal cardiac rhythm;
- Adequate oxygenation on FiO_2_ of ≤ 0.4 to maintain SaO_2_ ≥94%;
- Peak inspiratory pressure (PIP) ≤ 20 cmH_2_O;
- Maitenance pH > 7.35 and PaCO_2_ < 45mmHg with PEEP ≤ 6cm H_2_O and pressure support ≤ 6 cmH_2_O for at least 30 minutes;
- The level of consciousness consistent with adequate airway protective reflexes, absence of accessory respiratory muscle recruitment.

**Prophylactic CPAP Criteria**

The patients considered at high risk for extubation failure included:

- Patients receiving prolonged continuous invasive mechanical ventilation;
- Patients undergoing a complicated cardiac surgery with RACHS-1 score of 4 to 6;
- Evidence of cardiac insufficiency, or with low cardiac output at the time of extubation;
- Evidence of high risk of respiratory tract obstruction, such as laryngomalacia, bronchopulmonary dysplasia, pulmonary edema, acute lung injury or acute respiratory distress syndrome;
- Children with a neuromuscular disorder, and children with suspected/proven diaphragm dysfunction;
- Children with severe malnutrition.

**Reintubation criteria**

The possible reasons for reintubation included:

- Clinical signs of respiratory fatigue and severe respiratory distress despite maximum CPAP support;
- CPAP intolerance (due to difficulty tolerating the nasal or facial mask or lack of cooperation) ;
- Worsening hypercarbia (increase of ≥20 % from the baseline value) or hypoxemia (decrease of ≥20 % from the baseline value) ;
- Inability to clear airway or oral secretions;
- Hemodynamic decompensation, cardio-respiratory arrest;
- Glasgow scale ≤ 8 or inability to maintain adequate airway patency due to neurologic impairment.
