# Supplementary material for: The role of plasma gelsolin in cardiopulmonary bypass induced acute lung injury in infants and young children: a pilot study
Source: BMC Anesthesiol. 2014 Aug 7;14:67. doi: 10.1186/1471-2253-14-67 (PMC4132929; doi:10.1186/1471-2253-14-67)
Supplement: Additional file 2 — Definitions of clinical variables. [file 1471-2253-14-67-S2.doc]

**Additional File 2: Definitions of clinical variables**

**RACHS-1:** Risk adjustment for surgery for congenital heart disease: the RACHS method were six risk levels used to adjust for baseline risk differences and allow meaningful comparisons of in-hospital mortality for groups of children undergoing congenital heart surgeries, detailed in article by Jenkins KJ, et al.

**Nosocomial pneumonia (NF):** A documented source of infection in lungs by a positive microbiological culture, fever exceeding 38.5℃ or less than 35℃, white cell count exceeding 12×10^9^ or less than 4×10^9^.

**Positive fluid balance after CPB:** Positive fluid leaving in body at the end of CPB.

**EF**: Extubation failure (EF) was defined as the reinstitution of mechanical ventilators support (including noninvasive mechanical support) within 24 hrs after extubation.

**Duration of mechanical ventilation:** The total duration of invasive and noninvasive mechanical ventilation.
